# A near-natural experiment on factors influencing larval drift in *Salamandra salamandra*

**DOI:** 10.1038/s41598-022-06355-9

**Published:** 2022-02-28

**Authors:** Malwina Schafft, Norman Wagner, Tobias Schuetz, Michael Veith

**Affiliations:** 1 Department of Biogeography, Trier University, Universitätsring 15, 54296 Trier, Germany; 2 Department of Biology and Ecology of Fishes, Leibniz-Institute of Freshwater Ecology and Inland Fisheries, Müggelseedamm 310, 12587 Berlin, Germany; 3 Zweckverband Natura Ill-Theel, In der Meulwies 1, 66646 Marpingen, Germany; 4 Department of Hydrology, Trier University, Behringstraße 21, 54296 Trier, Germany

**Keywords:** Ecology, Ecology, Hydrology, Limnology, Animal behaviour, Zoology, Herpetology

## Abstract

The larval stage of the European fire salamander (*Salamandra salamandra*) inhabits both lentic and lotic habitats. In the latter, they are constantly exposed to unidirectional water flow, which has been shown to cause downstream drift in a variety of taxa. In this study, a closed artificial creek, which allowed us to keep the water flow constant over time and, at the same time, to simulates with predefined water quantities and durations, was used to examine the individual movement patterns of marked larval fire salamanders exposed to unidirectional flow. Movements were tracked by marking the larvae with VIAlpha tags individually and by using downstream and upstream traps. Most individuals showed stationarity, while downstream drift dominated the overall movement pattern. Upstream movements were rare and occurred only on small distances of about 30 cm; downstream drift distances exceeded 10 m (until next downstream trap). The simulated flood events increased drift rates significantly, even several days after the flood simulation experiments. Drift probability increased with decreasing body size and decreasing nutritional status. Our results support the production hypothesis as an explanation for the movements of European fire salamander larvae within creeks.

## Introduction

Organisms living in lotic waters are constantly exposed to unidirectional currents. Their movements can therefore be either passively drifting downstream (drift) or actively moving downstream or upstream. Passive downstream drift has often been studied for invertebrates^1–4^. The identified biotic and abiotic factors that determine the intensity of drift in invertebrates are current, flow rate, light conditions, water chemistry, temperature, food availability, population density and predator pressures^1,4^.

To cope with the adverse effects of downstream drift, organisms have evolved various strategies that either avoid drift in advance or compensate drift that has already occurred. The avoidance of downstream drift can be achieved through morphological or behavioral adaption. A variety of morphological adaptions are known from insect larvae, such as a flattened body or hooks, claws (e.g. Ephemeroptera) and suckers (e.g. Diptera); the latter enhance the ability to adhere to surfaces^5^. Possible behavioral adaptions include hiding under stones or other shelters (e.g. wood debris) within a stream or the preference for slow-flowing or even almost lentic microhabitats, such as potholes. Some species have even evolved positive rheotaxis, a further behavioral adaption that describes the propensity of an organism to constantly move against the water current^6,7^. If, however, a downstream drift has occurred despite the respective avoidance strategy, it can finally be compensated either by an a priori excess production of offspring in upper parts of a creak or stream (‘production hypothesis’^3^) or by upstream movement, which is usually carried out by mature or at least older life stages (‘colonization hypothesis’^1^).

Adaption to the unidirectional flow of lotic waters is also known from stream-dwelling amphibians. Some lotic anuran larvae show enlarged oral discs or belly suckers to adhere to submersed surfaces^8^. However, there is also a general evolutionary trend towards a flattened body shape in lotic anuran larvae^9^. Adaptions of salamander larvae to lotic environments often encompass a flattened body shape with lower tail and body fins^10^. Such morphological adaptations often enable the species not only to avoid drift but also to actively move upstream. In some stream-dwelling salamanders, upstream movements even prevail^11–13^. In the northern two-lined salamander, *Eurycea bislineata,* the downstream drift of the larvae of the first year is in part compensated by the upstream movement of second-year larvae, which supports the colonization hypotheses^14^. In contrast, Cecala, et al.^11^ concluded that neither the production nor the colonization hypothesis fully explains stream movements of larval salamanders.

The European fire salamander, *Salamandra salamandra*, is a larviparous species in which the females give birth to fully developed larvae^15^. It is the only Central European amphibian species that deposits its offspring in lotic waters, predominantly in first and second order streams^15,16^. Due to the non-selective deposition of the larvae by the females^17^, the larvae are often drifted downstream^15,16^ and accumulate in more lentic potholes^18^. However, European fire salamander larvae seem to lack morphological adaptations to their lotic environment, as known from other stream-dwelling salamanders^18^. Hence, the larvae should rather show behavioral adaptations^19^. Within creeks they are mainly found in areas with low flow velocity^18^. In addition, they show positive rheotaxis and move actively upstream^20^. However, such upstream movements are rare. The ratio of upstream–downstream moving European fire salamander larvae ranged from 2.6% to 11.2% per creek, indicating that upstream movements were not sufficient to compensate for downstream drift^20^. Furthermore, no difference in total body length between upstream and downstream moving larvae were found, while downstream drifting larvae were smaller than non-drifting larvae^20^. Thus, Veith et al.^20^ interpreted the observed upstream movements as an individual response to the water flow and probably as a side effect of the larvae’s propensity to avoid downstream drift^20^.

As a result, downstream drift seems to be the predominant movement of European fire salamander larvae in the lotic environment^20^. Its detrimental impact on the larval population has been demonstrated in several studies^16,18,21^, because once they have drifted downstream into the brown trout region, the predation risk increases to 100%^16^. Thiesmeier and Schuhmacher^16^ classified the larval drift of European fire salamanders into three categories: new-born drift, behavioral drift and catastrophic drift. Behavioral drift is caused by insufficient resource availability. Larvae that have to actively search for food or shelter are at risk of being caught by the current and to be drifted downstream. Catastrophic drift is caused by increased flow velocity due to heavy rainfalls. Increased water flow increases the number of drifted larvae^21^. Together with debris up to 90% of the larvae can be flushed downstream^22^.

Reinhardt et al.^17^ recently analyzed drift in the European fire salamander by tracking the spatial and temporal fate of individually marked larvae in three German first-order streams. While they confirmed the catastrophic effect of high summer precipitation as a trigger for larval drift, they could not find a significant evidence for random drift. In contrast to previous studies (e.g.^16^) they found neither a significant influence of body length nor body mass on the probability of a larva drifting downstream.

Here we investigate the individual up- and downstream movements of European fire salamander larvae within a near-natural creek, with the possibility of experimental manipulation of the water flow. This experimental setting has important advantages over previous studies conducted in the laboratory (e.g.^16,17,21^) or in natural systems^17^. Our closed system enabled us to keep the water flow constant over time and, at the same time, to induce flood simulations with predefined water quantities and durations. This allowed us to study both behavioural and catastrophic drift in the same experimental setup. The maximum population size was known throughout the experiment and, in contrast to Reinhardt et al.^17^, we were able to calculate population-specific daily drift rates using recapture rates of individually marked larvae. This allowed us to apply a multivariate approach to test the following predictions:

1. Drift rates measured before a flood simulation experiment are lower compared to those measured after such an experiment (e.g.^17,20,23^); this corresponds to the catastrophic drift hypothesis^4^.
2. The overall drift rate measured during the period of discharge manipulation remains higher than before; this assumption is based on the observation that not only the salamander larvae but also their prey organisms are negatively affected by catastrophic drift (e.g.^24^); the behavioural drift of the larvae therefore often starts with a time delay during the subsequent foraging and thus only after the floodwater has receded^23^.
3. Larvae actively foraging are likely to get caught in the current^24^; therefore we further assume that drifted larvae have a poorer nutritional status than non-drifted larvae.
4. Drifted larvae are smaller than non-drifted larvae because large larvae can better resist water current^16,20^ (but see^17^) and can survive starvation periods longer^25^.
5. Drift rates increase with increasing water temperature; this has generally been demonstrated for invertebrates^3,4^ and should also apply to heterothermic vertebrates; in Central Europe, the headwaters of first and second order streams, the preferred larval habitat of European fire salamanders, carry nutrient-poor and cool spring water with slightly fluctuating average annual temperatures of mostly 8–10 °C^26^. As with invertebrates, increased temperatures should therefore cause stress; larvae seeking cooler shelters to escape the higher temperatures may be caught in the water current.

## Materials and methods

To assess upstream and downstream movements in the artificial creek, we caught 100 larvae (under license of the ‘Struktur- und Genehmigungsdirektion (SGD) Nord’ of the Federal State of Rhineland-Palatinate, Germany; permit number 425-104.1706) between mid-May and mid-June 2015 in a first-order creek (Beresbach, Federal State of Rhineland Palatinate, Germany) depending on their availability. Each individual was marked with fluorescent Visible Implant Alphanumeric (VIA) tags (size 1.2 mm × 2.7 mm) of the Northwest Marine Technology Inc. (see Wagner et al.^27^). The tags were injected at the base of the tail on the right side of the body. This treatment was approved by the Ethics Commission of Trier University and carried out in accordance with the relevant animal welfare guidelines and regulations of the Federal State of Rhineland-Palatinate. All methods are reported in accordance with the ARRIVE guidelines. Two different colours (neon-yellow and neon-orange) were used to facilitate differentiation of two experimental cohorts (see below). After tagging, the larvae were kept in aquaria in a climatic chamber at 15 °C for one week and fed with macro-invertebrates from a nearby creek before being put into the artificial creek in order to ensure tag retention.

**Figure 1:**
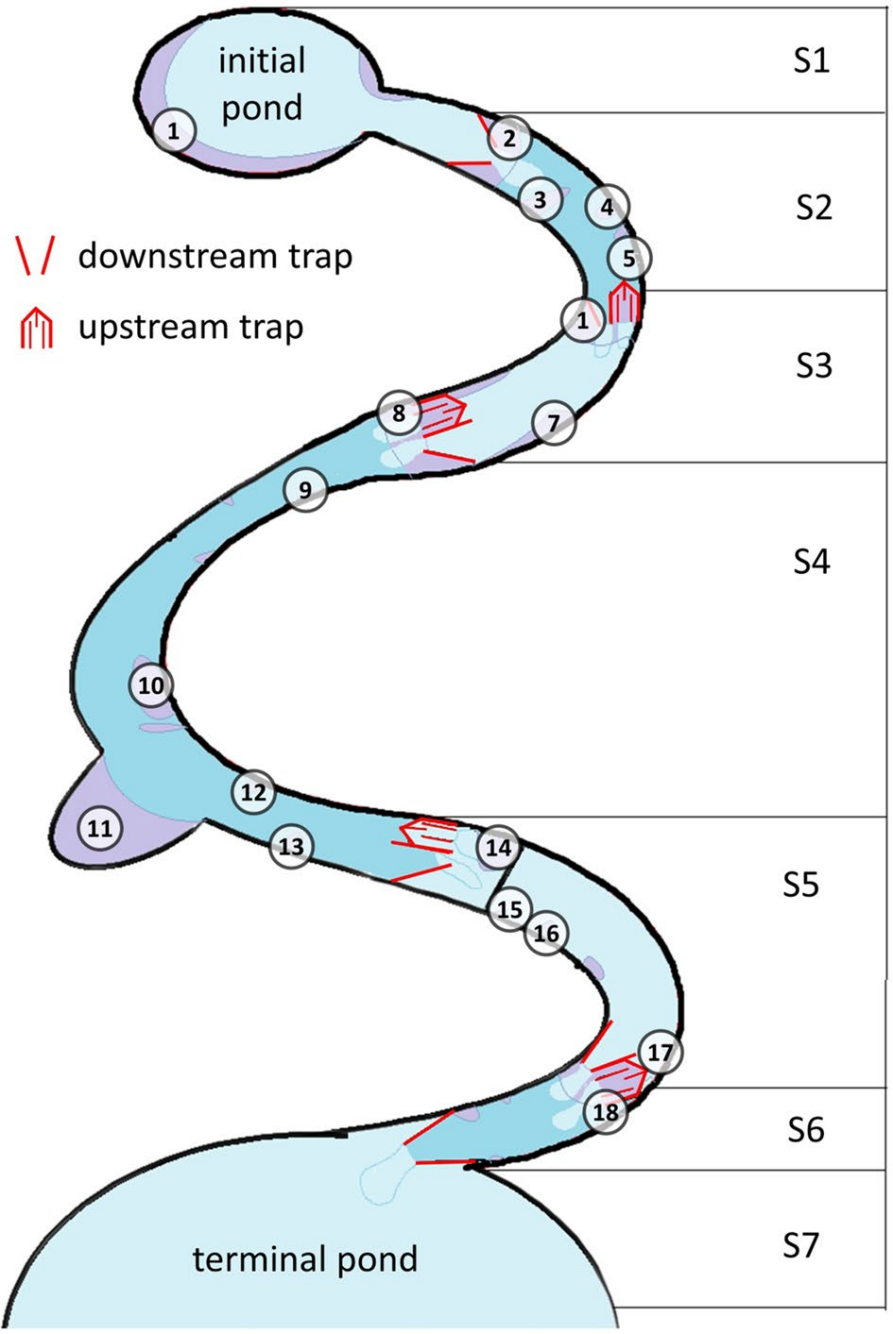
Schematic representation of the study design in the near-natural artificial creek. Downstream and upstream traps divided seven sections (S1-S7). The colours indicate areas of low (purple), medium (light blue) and high (dark blue) flow velocity. The introduction sites are marked by numbers.

The artificial creek originated in a small initial pool from where the water flowed down in three bends over a total length of 32.5 m into a terminal pond (Fig. 1). From here the water was pumped back into the initial pool, forming a closed circuit. The creek bed was made of black pool liner and filled with gravel and chalk-sandstone rocks. In the second bend there was a permanent pool. Between the second and the third bend there was a small waterfall of about 30 cm height. Since European fire salamander larvae are mainly found in places with low water velocity^18^, 16 additional potholes were formed with gravel and stones to create small areas of reduced flow velocity. Slates of different sizes were added to provide hiding places. The slates also facilitated the search for larvae in hiding places. The widest point of 2.1 m was in the second bend at the pothole. Apart from that, the widest place of 70 cm was between the first and the second bend and the smallest place of 30 cm was directly behind the initial pool. The difference in height from the initial pool to the pond at the end of the creek was about 5–6 m. The inclination measured at randomly selected points within the course of the creek ranged from 0 to 58%. On average, the inclination was 18%; the upper part of the creek has a comparatively lower inclination than the lower part.

**Figure 2:**
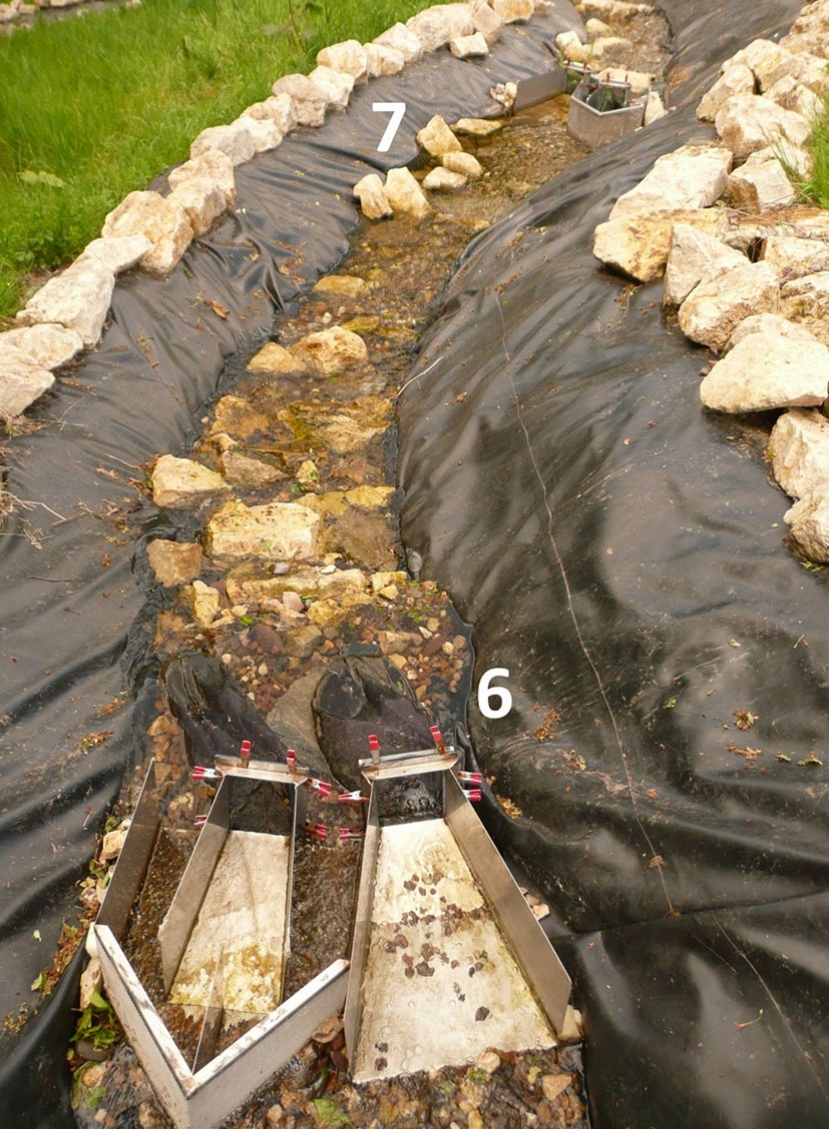
Two pairs of upstream and downstream traps. They delimit section S3. Introduction sites 6 and 7 are indicated.

Two different types of traps were installed at different places within the creek (pairwise if possible) to record the movements of the larvae. Each pair consisted of a downstream and an upstream trap, which together completely blocked the creek bed (for details see Veith et al.^20^). In narrow places only the downstream trap fitted into the creek bed. This was the case at the beginning of the creek directly behind the initial pool and at the end of the creek directly in front of the pond. Four pairs of traps were installed in between (Fig. 2). Water temperature was measured every hour by three data loggers (Tinytag loggers of Gemini Data Loggers UK Ltd., Chichester, UK), which were put beneath stones within the stream to not being exposed to direct radiation.

After weighing (to the nearest 0.001 g using a Kern EMB 200-3 field balance) and photographing on graph paper (to later determine the snout-vent-length, SVL, as the distance between the tip of the snout and the hind legs as well as the head width to the nearest 0.5 mm using the software Datinf®^23^), the larvae were randomly introduced at one of the 18 introduction sites (initial pool, permanent pothole and additional potholes modelled with slate). Of the 100 initially tagged larvae, 82 were finally introduced into the experiment (twelve lost their tag before introduction, and six developed signs of a beginning metamorphosis after being tagged). Depending on availability from the source creek, the first 18 larvae were introduced into the experiment on May 11, followed by two larvae on May 26, nine larvae on June 2, one on June 5, nine on June 9 (up to this date all larvae had yellow tags), 17 on June 16, ten on June 18 and 16 on June 19 (all these larvae had orange tags). In order to ensure sufficient feeding of the larvae, the artificial creek was stocked ten times with macroinvertebrates before the first larvae were introduced. The stocking was repeated once a week throughout the trial period. During the period of flood simulation experiments, the invertebrate stocking was done one day after each flood simulation experiment.

The water flow of the artificial creek was kept constant at a rate of 48 l/min. Towards the end of the experiment, four flood simulations were initiated (June 30, July 4, July 8 and July 12). To simulate flood peaks, the pumping rate could be increased to 192 l/min; additional water could be pumped into the system from a 1,000 L water tank at the same maximum flow rate of 192 l/min. Two different types of flooding were simulated to test different stress situations caused by increased water flow. We first simulated a continuous but gentle increase in flow rate within the first 10 min, followed by a constant flood peak of 384 l/min for 70 min. After that, the circulating water flow was kept constant at 192 l/min until the next morning and then dropped to the regular 48 l/min. This was to simulate an increased water flow, e.g. after a thunderstorm. The second type of flood simulated an irregular and potentially more stressful flow of water; it started directly with the maximum flow rate of the first pump (192 l/min) and simulated another sudden increase in discharge (in less than a minute) towards 384 l/min, with two short interruptions of lower discharges (192 l/min) for five minutes each. Again, the circuit water flow was then kept constant at 192 l/min until the next morning, when it dropped to the regular 48 l/min.

The entire experiment took place from May 11 to July 15. The traps were inspected for larvae twice a week. During the flood simulations traps were checked every other day. The larvae were counted as either moving downstream (*N*_*d*_) or moving upstream (*N*_*u*_). VIA tags were read with the help of UV light, weight and SVL were measured at each inspection event as described above. Larvae with yellow tags were returned randomly to one of the 18 introduction sites (‘random treatment’); this ensured that data could be collected across the entire artificial creek. Larvae with orange tags were released at the closest introduction site in the direction in which they had moved (‘directional treatment’): below a downstream trap or above an upstream trap. This directional treatment was designed to mimic movement histories that were not interrupted by our treatment. To gather information from a non-captured control group, we searched for larvae in the introducing sites between the traps after every third trap control event (*N*_*s*_). They were identified and measured as described above to obtain representative samples of body size and nutritional status and subsequently returned to their pothole of capture. To ensure that our search for larvae at the introduction sites had not unintentionally induced additional up and down movements, the traps were then checked again for larvae. Larvae that entered metamorphosis or had lost their tag were removed from the experiment. The nutritional status of all larvae was calculated as Scaled Mass Index (SMI)^28^.

Prior to estimating population size, we tested if the two reintroduction treatments of trapped larvae might have caused a heterogeneity in their capture probability. We used the subroutine ‘closed captures’ in MARK^29^ to compare the probabilities of the models of^30^ including group-specifity: g*M_0_ (equal capture probabilities), g*M_t_ (time-dependent capture probabilities), g*M_b_ (behavioural effects on capture probabilities), g*M_h_ (group-specific heterogeneity in capture probabilities) and combinations thereof. We used only capture histories of trapped larvae, since non-trapped larvae were not exposed to different reintroduction treatments. We expected a model including group specific capture probabilities (g*M_h_, g*M_bh_, g*M_th_ or g*M_bth_) to be the best model if the two reintroduction groups would differ in their capture probabilities. The Otis et al.^30^ models were compared with or without group specific capture probability. Model selection was based on AICc values.

We estimated population size in order to be able to calculate drift rates for each control day. We used the software MARK also to estimate the population size for each control event. We calculated the survival probability (*Φ*), the capture probability (*p*) and the entering probability (*pent*) using the subroutine POPAN^31^, which provides a Cormack-Jolly-Seber (CJS) estimate for open populations^32–34^. We selected the best fitting population model using the AICc criterion. To see how well the estimated population sizes reflect reality, we determined the minimum and maximum possible population sizes for each control event. The minimum value for a given control day was obtained by adding the number of individuals actually caught on that day and the number of individuals not caught at this day but on later days. The maximum value was calculated by subtracting dead or removed individuals from the number of larvae introduced into the system prior to this capture day.

The data were tested for normal distributions using the Shapiro–Wilk test^35^. Depending on the result of the Shapiro–Wilk test, drift rates were compared using a paired t-test for hypothesis I, the unpaired t-test for Hypothesis II, III and IV and the Wilcoxon-Mann–Whitney test for non-normally distributed data for hypotheses V. To test hypotheses V, drift rates were correlated with water temperature (average per interval between two trap control events across all three loggers) using the Spearman’s rank correlation; for this test, we had to confine to the data prior to the start of water flow manipulations in order to avoid that the then drastically increased water flow would mask potential temperature effects.

To account for co-variation of parameters, we performed a generalized linear mixed model (GLMM). We included SMI, head width, water temperature and treatment group as independent variables and analyzed them by stepwise AIC to identify the main factors that influence larval drift as binomial response variable. In addition, we used ID of larvae and date of measurement as random effects to account for variation in individual behavior and for multiple drift events with the same temperature due to same sampling interval. GLMM was performed using the glmer function of the lme4 package in R^36,37^. Statistical tests were also performed using the software packages R^37^.

## Results

During the 22 capture occasions, 69 drift events (34 in the directional treatment, 22 in the random treatment and 13 unidentified larvae) were found in downstream traps. Only three times a larva was found in an upstream trap (one in the directional treatment, two in the random treatment), so that further analysis of the factors affecting upstream movements is not possible. 132 times larvae were caught in one of the 18 introduction sites.

Four larvae were found dead in the downstream traps. 31 individuals drifted at least once, three individuals moved upstream, and 33 individuals were caught only outside traps. 15 individuals were never caught after their release. 19 individuals drifted only once, two individuals drifted twice, five individuals three times, three four times, while two individuals drifted up to five times. Observed distances of upstream movements did not exceed 30 cm. The longest single drift event covered a distances of 10.5 m (maximum distance between two traps). The larvae drifted up to four times in a row.

**Figure 3:**
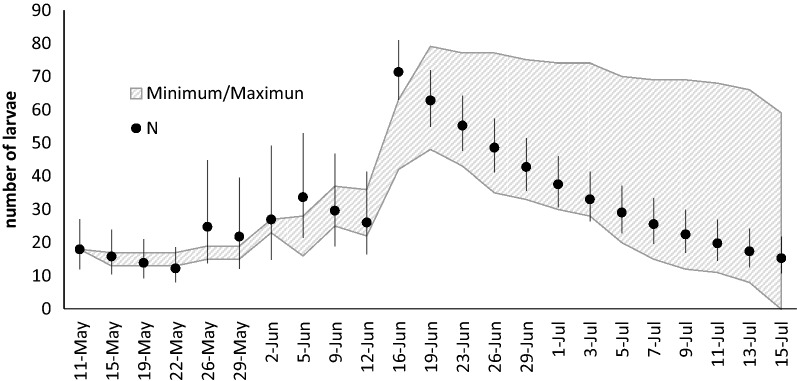
Population size estimate for each date with 95% confidence interval. Mean estimate (N) as black circles is shown with the calculated possible range (crosshatched area). Four values of the population estimates are higher than the possible range of values. But as the possible range still is contained in the 95% confidence interval of these four cases, the highest possible value has been used for the calculation of drift rates.

Our test for heterogeneity of capture probabilities among trapped larvae (‘closed captures’ in MARK) showed two models to be almost equally probable (g*M_tb_ and g*M_tbh2_, with AICc being 203.6524 and 203.6525, respectively; see SI). This clearly shows that temporal and behavioural effects on capture probability exist. Adding group-specific heterogeneity to the model does neither increase nor decrease model probability. Therefore, we decided to estimate population size in MARK for both treatment groups together (now including capture probabilities of trapped and not trapped larvae). The best population size model (AICc = 926.3; see SI) was estimated with time and group dependent parameters *Φ*, *p* and *pent*. ΔAICc of the second-best model was 17.9. In the beginning, the overall population size was estimated at 18 individuals and increased until the last introduction of additional larvae at June 19. After reaching a maximum of 63, the population size declined to 15 towards the end of the experiment. Between May 25 and June 2 and June 16, MARK estimated population sizes larger than the possible maximum (see Fig. 3). As the maximum possible population size was included in the 95% confidence intervals of the estimate, we corrected the population size to the highest possible values for the calculation of daily drift rates. For all other days we used the estimated population size for drift rate calculation.

**Figure 4:**
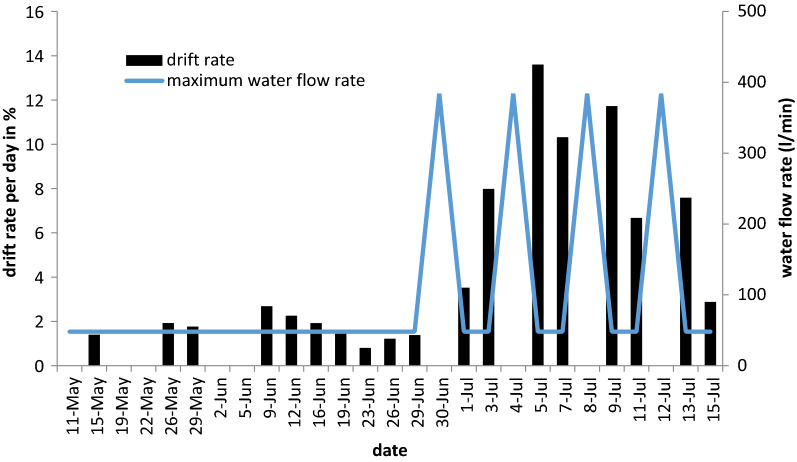
Drift rates per day and maximum water flow rate per day over the trial period. High maximum water flow rates indicate the dates of experimental flood simulations.

Daily drift rates ranged from 0 to 2.7% (mean value: 1.2%) before the flood simulations. After the first simulation, they increased, with drift rates then ranging from 2.9 to 13.6% (mean value: 8.02%; see also Fig. 4). The drift rates measured after flood events with mean 9.1%) were significantly higher than those measured directly before the respective simulation with mean 6.6% (H I: one-sided paired t-test: t = 2.37, df = 3, p-value = 0.049, Table 1). Drift rates during the manipulation period remained significantly higher even in the intervals without flood simulations with mean drift rate of 7%, than in the period before flood simulations started with a mean of 1.2% (H II: one-sided t-test, t = 3.65, df = 3.15, p-value = 0.02, Table 1).

**Table 1:** Sample size (N), mean and standard deviation (M (SD)) with test statistics for each hypothesis.

Hypothesis	Measure	Before		After		p-value	Test
		N	M (SD)	N	M (SD)		
H 1	Drift rate per day in %	4	6.6 (3.8)	4	9.1 (4.5)	0.049*	Paired t-test
H 2	Drift rate per day in %	14	1.2 (0.9)	4	7 (3.1)	0.02*	t-test

		Drifted		Non-drifted			
		N_d_	M (SD)_d_	N_c_	M (SD)_c_		
H 3	Scaled mass index (SMI) in g	65	0.8 (0.14)	123	0.89 (0.15)	< 0.01*	Mann–Whitney U-test
H 4	Body sizes as head width in mm	65	7.8 (0.5)	123	8.1 (0.8)	< 0.01*	t-test

				N	r		
H 5	Temperature and drift/day			14	0.4	0.16	Pearson correlation

*Significant < 0.05.

The scaled mass index (SMI) was calculated with obtained b_sma_ = 3.229 and L_0_ = 24.76 mm (arithmetic mean of SVL). It was significantly lower for drifted larvae (N_d_ = 65, mean = 0.8, SD = 0.14) than for non-drifted larvae (N_c_ = 123, mean = 0.89, SD = 0.15) (H III: Mann–Whitney U-test, W = 5567 p < 0.01, Table 1). SVL and head-width were highly correlated (Spearman’s rank correlation, rho = 0.89, p < 0.01), with head-width showing higher measurement accuracy. Therefore, we used the head width to compare the size of drifted (N_d_) and non-drifted larvae (N_c_). Head width was significantly lower in the drifted larvae (H IV: t-test, t = 3.92, df = 175, p < 0.01; N_d_ = 65, mean_d_ = 7.8, SD_d_ = 0.5; N_c _= 123, mean_d_ = 8.1, SD_d_ = 0.8, Table 1).

The mean water temperature during the test period was 18.02 °C. It ranged from 11.29 °C (on May 21) to 29.51 °C (on July 4). Before the flood simulation experiments no significant correlation between drift rates and temperature could be found (Pearson correlation, r = 0.4, p > 0.1, Table 1).

A generalized linear mixed model (GLMM) using drift rate as response variable and scaled mass index (SMI), head width, temperature and reintroduction treatment (random vs directional) as fixed effects and ID of larvae and date of measurement as random effects showed that the model including SMI and head width as fixed effects fits best (AICc = 168.4 (Table 2). Although fixed effects were not significant and marginal R^2^ therefore only 0.13, the model including SMI and head width as fixed effects was still the best model fit. The drift probability increases with decreasing head width and SMI. Most of the variance was explained by random effects with conditional R^2^ = 0.85. Most of the variance was explained by date of measurement as one of two random effects.

**Table 2:** Generalized linear mixed model with drift as binomial dependant variable.

Random effects	Groups	Variance	Stand.dev	p-value
ID	66	6.77	2.6	< 0.01
date	20	12.31	3.5	< 0.01

Fixed effects	Estimate	Standard error	z-value	p-value
Intercept	− 0.27	1.07	− 0.25	0.8
Head width	− 1.41	0.93	− 1.52	0.13
SMI	− 1.16	0.67	− 1.73	0.08
Marginal R^2^	0.13			
Conditional R^2^	0.85			

Individual ID and date as fixed effects, and head width with scaled mass index (SMI) as independent variables obtained the best AIC of 168.4. Marginal R^2^ (associated with fixed effects) is low, while conditional R^2^ (associated with fixed effects plus random effects) is high.

## Discussion

Our study is the first that analyzes factors that were mentioned as potential triggers of drift in larvae of the European fire salamander simultaneously and under controlled near-natural experimental conditions. It takes advantage of the possibility to induce floods with known flow velocities and to relate the number of drifted individuals to population sizes estimated for an open population.

We found that experimental flood simulations caused increased drift rates that remained high even in following intervals without flood simulations (hypotheses I and II), but in contrast to our expectation, stress caused by high temperatures did not affect drift rates (hypothesis V). Temperature might not be such an important stressor that forces larvae to leave their shelter. Indeed, larvae were found to show high site fidelity, and upstream movements did not exceed more than 30 cm. In general, our findings do, however, indicate that drift occurs mainly during floods. Yet we also observed drift events during low water flow velocity and mainly small larvae and larvae with low nutritional status were affected. Altogether, effects of flood simulations were very clear, while impact of size and nutritional status were less consistent.

Drift is an important factor influencing the density of larvae of the European fire salamanders in lotic environments^16^. On average, 30% of all larvae drift downstream, whereby mainly newborns are affected^23^. Behavioural drift avoidance has been observed both in experiments^21^ (for the congeneric *S. infraimmaculata*^38^) as well as in nature^18,20^. Body size^20^, nutritional status^23^ and intraspecific aggression^25^ may influence the strength of drift. In our near-natural experimental setup and on the basis of individual capture histories, we quantified the strength of flood-induced drift. We also showed that, in addition to floods, body size and nutritional status (SMI) actually have a significant influence on the drift rates of European fire salamander larvae. Small size and low SMI fostered larval drift. Both can be seen as indicators of stress, with SMI being the consequence of an insufficient food availability or a low food intake due to a competitive disadvantage, e.g. because of a small body size.

The results of the GLMM showed that impacts of nutritional status and size of larvae was less strong when individual behavior of larvae and date of measurement were considered as random effects. Larvae that entered the system late tended to have a lower body size and lower nutritional status than larvae already in the system. These individuals therefore were less competitive and drifted more often. These multiple drift pattern by small and not well nourished larvae may explain the high impact of ID in the model and mask the effect of SMI and head width. The high impact of date of measurement can be explained by high drift rates as soon as flood simulations started. Temperature and the two reintroduction treatments did not influence the drift probability.

In nature, flood events immediately and permanently increase drift of the larvae^16^. The possibility of triggering flood events in the artificial creek enabled us to study their effect under experimental conditions. The controlled increase of the flow rate facilitated the determination of the maximum discharge without compromising the function of traps, since the increase was stopped as soon as first small stones in the creek bed started moving. In natural systems, floods can lead to a high transport of debris^22^ and thus further increase the drift rate of larvae. In comparison, the flood events simulated by us can be considered moderate. The daily drift rates increased significantly after flood events, showing that the drift was directly induced by our flood simulations. This corresponds to observation from the field about the immediate effect of drift, namely catastrophic drift, in European fire salamanders^16^.

However, after the water flow of our artificial creek was manipulated for the first time, the drift rate remained high or even increased in intervals without a flood simulation. It averaged 1.2% before the start of the flood simulations, but increased to an average 7% after the simulations and remained high even three days after a flood event. Such delayed drift rates after flood events were also seen in the field^16^. It was observed that outside the time of birth most drift events did not occur during the highest water levels, but a few days later. The authors explained this by a changed behavior of the larvae, probably induced by a reduced food availability and the necessity for larvae to move around to find food (for a detailed discussion see^15^). In addition, persistent disturbance of microhabitats, especially shelters, may have also contributed to increased larval mobility and hence to an increased drift rate.

Larvae of *S. salamandra* are ‘sit-and-wait hunters’, so they are usually highly stationary within a stream^16^. This strategy minimizes the risk of being flushed downstream by the current. Larvae of other urodelan species living in similar habitats follow the same strategy (e.g., the Pyrenean brook newt, *Calotriton asper*^39^). However, Thiesmeier and Schuhmacher^16^ have shown that the larvae leave their shelter more often when hungry. Therefore, a low nutritional status (as reflected in the SMI) increases the likelihood of drift. In addition, individuals with a low nutritional status may be weaker and therefore less able to resist the water current. This adverse effect can be even stronger during phases of increased discharges in natural habitats (so-called catastrophic drift^4,22^) or, alternatively, during the flood simulations in our experiment.

It is known that both abundant food and high temperatures lead to rapid growth of salamander larvae^19,40^. Fast growing larvae can soon become strong enough to resist the water velocity and thus to minimize drift. Our data clearly show that under the near-natural conditions of our experiment the probability of drift decreased with increasing head width. Bigger larvae can be found in places within a stream with higher flow velocity than smaller ones^18^. Bigger larvae can also cope better with higher water flow velocities within a glass tube than smaller larvae^21^. In addition, smaller larvae are exposed to more intraspecific competition^25^ (also in the Near Eastern fire salamander, *S. infraimmaculata*^41^) and may be forced to leave their refuges more often, so they can be more easily captured by the current. Due to their insufficient ability to withstand the current, they may drift downstream more often than larger individuals exposed to the same conditions.

The water temperature in the artificial creek was higher than the water temperature of natural creeks that in our area are usually inhabited by larvae of the European fire salamander. Their natural habitats are predominantly first order creeks inside forests^42^, which are characterized by comparatively low water temperatures all year round^43^. From mid-June to mid-July 2017, the water temperature in the Beresbach from which our larvae originated fluctuated between 11.6 and 18.0 °C (data not shown). This is considerably lower than the temperatures measured between mid-June and mid-July in our artificial creek (17.7–25.9 °C). The lack of shading trees in combination with the water flowing in a closed circuit and the hot weather during the trial period in summer 2015^44^ certainly contributed to increased water temperatures at our experimental site. It has been shown that larvae of *S. infraimmaculata* from Israel move towards favorable water temperatures^45^, with a temperature optimum between 10 and 20 °C. For larvae of the European fire salamander, temperatures below 6 °C and above 23 °C can have harmful effects^40^. We did not observe upstream or downstream movements that were related to increased water temperature. This might result from a bigger scale of our experiment, and we could therefore not observe small scale movements of the larvae. Another explanation could be that our artificial stream did not offer more suitable temperatures to move to. Further we interpret this result that temperature as a stressor does have less impact on larval movement than floods, food availability and intraspecific competition.

## Conclusion

Several factors influence the drift of European fire salamander larvae. One intrinsic factor is body size (which determines a larva’s ability to resist the current), while extrinsic factors are food availability (which affects a larva’s the nutritional status) and discharge rates (e.g. during flood events). All factors act simultaneously on larvae in lotic environments, but their joint effects and interactions have never been analyzed in a multivariate approach.

In natural habitats, the highest drift rates are observed in newborn larvae^16^. However, once the larvae have established themselves in a creek and found shelter, they remain stationary if the water flow is more or less stable and prey is abundant. This behavior, in combination with an overall positive rheotaxis^20^, can be considered an adaptation to avoid downstream drift. Since many brook-dwelling salamanders show stationarity or dominance of upstream over downstream movement, Cecala, et al.^11^ assumed that neither the colonization hypothesis^1^ nor the production hypotheses^3^ sufficiently explained the behavior of salamander larvae in creeks. However, for the larvae of the European fire salamander, downstream drift is by far the dominant mode of movement^20^. Upstream movements are rare events and are not sufficient to compensate for losses of individuals due to downstream drift. Our results, based on individual capture histories, controlled flooding events in a near-natural creek and the consideration of covariation of predictor variables, therefore clearly show that drift is indeed predominantly caused by extrinsic factors. This further supports the production hypothesis as an explanation for the movements of European fire salamander larvae within creeks.

## Supplementary Information

Supplementary Information.
